# Myelin Defects in Niemann–Pick Type C Disease: Mechanisms and Possible Therapeutic Perspectives

**DOI:** 10.3390/ijms22168858

**Published:** 2021-08-17

**Authors:** Antonietta Bernardo, Chiara De Nuccio, Sergio Visentin, Alberto Martire, Luisa Minghetti, Patrizia Popoli, Antonella Ferrante

**Affiliations:** 1 National Center for Drug Research and Evaluation, Istituto Superiore di Sanità, Viale Regina Elena 299, 00161 Rome, Italy; antonietta.bernardo@iss.it (A.B.); sergio.visentin@iss.it (S.V.); alberto.martire@iss.it (A.M.); patrizia.popoli@iss.it (P.P.); 2 Research Coordination and Support Service, Istituto Superiore di Sanità, Viale Regina Elena 299, 00161 Rome, Italy; chiara.denuccio@iss.it (C.D.N.); luisa.minghetti@iss.it (L.M.)

**Keywords:** Niemann–Pick type C disease, myelination, oligodendrocytes, cholesterol, mitochondrial impairment, A_2A_R, adenosine

## Abstract

Niemann–Pick type C (NPC) disease is a wide-spectrum clinical condition classified as a neurovisceral disorder affecting mainly the liver and the brain. It is caused by mutations in one of two genes, *NPC1* and *NPC2*, coding for proteins located in the lysosomes. NPC proteins are deputed to transport cholesterol within lysosomes or between late endosome/lysosome systems and other cellular compartments, such as the endoplasmic reticulum and plasma membrane. The first trait of NPC is the accumulation of unesterified cholesterol and other lipids, like sphingosine and glycosphingolipids, in the late endosomal and lysosomal compartments, which causes the blockade of autophagic flux and the impairment of mitochondrial functions. In the brain, the main consequences of NPC are cerebellar neurodegeneration, neuroinflammation, and myelin defects. This review will focus on myelin defects and the pivotal importance of cholesterol for myelination and will offer an overview of the molecular targets and the pharmacological strategies so far proposed, or an object of clinical trials for NPC. Finally, it will summarize recent data on a new and promising pharmacological perspective involving A_2A_ adenosine receptor stimulation in genetic and pharmacological NPC dysmyelination models.

## 1. Introduction

Niemann–Pick disease belongs to the vast section of lysosomal storage disorders (LSD), which includes various inherited metabolic diseases caused by the deficiency of one of the different lysosomal functions [1]. The term Niemann–Pick disease refers to a group of diseases (type A, type B, and type C) whose common denominator is a genetically determined altered function of lysosomal proteins. Mutations in the *SMPD1* gene cause Niemann–Pick disease types A and B. They produce a deficiency in the lysosomal enzyme acid sphingomyelinase activity that breaks down the lipid sphingomyelin in ceramide and phosphorylcholine. Failure or insufficient functioning of this enzyme will result in an accumulation of sphingomyelin [2]. Niemann–Pick type C (NPC), on the other hand, is caused by the mutation in the *NPC1* and *NPC2* genes located on chromosome 18 (locus 18q11-q12) and 14 (locus14q24.3), respectively. The *NPC1* gene encodes a protein located in membranes of late endosome/lysosome compartment (LE/L) the *NPC2* encodes a protein that binds and transports cholesterol, and it has been shown to closely interact with NPC1. Genetic mutations in the *NPC1* gene cause approximately 95% of NPC cases, and 5% are caused by mutations in the *NPC2* gene. Mutations in both genes are responsible for a defect in intracellular transport of endocytosed cholesterol that results in the sequestration of unesterified cholesterol, sphingosine, glycosphingolipids, and sphingomyelin in the LE/L compartment of various tissues of the body, including the brain [3]. Transmission of the disease occurs in an autosomal recessive manner. NPC affects an estimated 1–9:100000 people (https://www.orpha.net/ accessed on 16 August 2021). It is classically a neurovisceral condition with a wide clinical spectrum. The principal clinical symptoms are hepatosplenomegaly, jaundice, and fetal hydrops with rapid death often due to hepatic and respiratory failure; other symptoms include hypotonia, delayed motor development, speech delay, cataplexy, cognitive impairment, dystonia, and psychiatric symptoms such as hallucinations and schizophrenia. Common signs of many patients are also ataxia, dysphagia, and vertical supranuclear gaze palsy [4]. NPC can arise at any age and a correlation exists between the age of onset and the severity of the disease; an early onset of clinical symptoms is associated, in fact, with a more rapid progression. At the cellular level, NPC is characterized by lysosomal accumulation of multiple lipids, such as sphingosine, glycosphingolipids, sphingomyelin, and especially cholesterol. The lysosomal accumulation of lipids and the subsequent inhibition of calcium uptake into the acidic compartment could result in an unbalance in autophagic flux, with an impaired fusion of late-endosomes and lysosomes [5,6]. Another classical feature of NPC is the accumulation of cholesterol in mitochondria. The increase in mitochondrial cholesterol can contribute to mitochondrial dysfunction and subsequent oxidative stress associated with the disease [7]. Furthermore, defective mitophagy and increased mitochondrial fragmentation could perpetuate mitochondrial dysfunction in NPC due to impaired mitochondrial turnover. Although all NPC cells show a defect in cholesterol accumulation, the major clinical impact is on the liver and brain. Cholesterol is particularly enriched in the brain, where it is involved in key biological functions, such as signal transduction pathways, myelin formation, and synaptogenesis. In the brain, the most severe consequences of *NPC1* mutations are neurodegeneration (due to the massive loss of Purkinje neurons in the cerebellum and the diffuse atrophy in other brain regions such as the hippocampus), neuroinflammation, and dysmyelination [8,9]. Herein, we will focus on myelin defects and the efforts made by research to identify and validate new therapeutic approaches to rescue from these debilitating events.

## 2. Cholesterol in Myelination 

Cholesterol is an essential component of the central nervous system (CNS) and the peripheral nervous system (PNS). In the CNS, it derives almost exclusively from the endogenous synthesis since circulating cholesterol is unable to cross the blood–brain barrier (BBB). Nevertheless, some lipoproteins, sterols, or other circulating molecules could enter the brain to deliver cholesterol [10]. About 80% of its brain content resides in myelin [11] where it is a key structural component necessary for the assembling and compacting of membranes so that the proper electrical insulation of neuronal axons is ensured [12]; cholesterol content influences also membrane fluidity, which in turn may influence myelin membrane spreading [12]. Indeed, its incorporation in myelin is rate-limiting for membrane growth [13].

Most of the cholesterol needed for the organization of myelin sheaths during postnatal myelination is synthesized by oligodendrocytes (i.e., the myelinating cells in the CNS, OLs) via the isoprenoid biosynthetic pathway [13,14]. Interestingly, cholesterol also regulates the expression of genes that encode myelin proteins necessary for the differentiation of OLs from progenitor cells, unable to form myelin leaflets, to mature myelinating OLs [15]. The molecular mechanism connecting cholesterol to gene expression is based on its ability to organize membrane microdomains necessary for signal transduction that promotes myelin gene expression [12]; in particular, the activity of the mechanistic target of rapamycin (mTOR) kinase, a major driver of myelination by OLs, requires cholesterol to induce myelin gene expression [12].

It is clear that cholesterol plays a key role in regulating OL functions and, consequently, the myelination process; this implies that a simple shift in the complex equipment of synthesis, assembly, or transport of this molecule is enough to activate pathologies of various orders and degrees. Table 1 collects some hypomyelinating diseases related to the altered functionality of cholesterol itself, considering three main events: biosynthesis, transport, or accumulation. For example, Smith–Lemli–Optiz syndrome, Peroxisome biogenesis disorder, Greenberg dysplasia, and Conradi–Hunermann syndromes are linked to mutations in genes coding for enzymes (7-dehydrocholesterol reductase—DHCR7, acyl-CoA oxidase 1-ACOX1, 3 b-Hydroxysteroid 8, 7-sterol isomerase) involved in the synthesis of cholesterol, and all four commonly lead to reduced myelin formation that clinically manifests itself in developmental delay, motor dysfunction, and more (for further details, specific references are indicated in the table).

For the focus of this review, particularly interesting is the observation that transport defects and intracellular accumulation of cholesterol are accompanied by myelin defects in many disorders. For example, the low-density lipoprotein receptor-related protein-1 (Lrp1), a member of the LDL receptor family with prominent functions also in endocytosis, regulates cholesterol homeostasis in OLs [16] and the differentiation of OL progenitor cells (OPs) [17]. This protein can be defective in some demyelinating diseases, such as peroxisome biogenesis disorders. In Pelizaeus–Merzbacher disease (PMD), missense mutations, duplications, and triplications in the *X-linked proteolipid protein (PLP1)* gene lead to hypomyelination, progressive loss of oligodendrocytes, and neurodegeneration. PLP1 associates with cholesterol to regulate its enrichment in myelin membranes [13,18,19]; its variants are misfolded and have a reduced ability to bind cholesterol; this causes their retention in the endoplasmic reticulum (ER) likely driving dysmyelination [15]. 

As mentioned in the introduction and as shown in Table 1, NPC is also conceived as a lipid trafficking disorder [20,21] in which mutations in both *NPC1* and *NPC2* genes are responsible for a block in intracellular processing of endocytosed cholesterol [20]. Both NPC1 and NPC2 are cholesterol-binding proteins, but the first resides in the membrane of LE/L, and the latter is found soluble in its lumen [22]. Functional studies have led to a “hand-off” model whereby cholesterol present in LE/L compartment is passed from the soluble NPC2 protein to the membrane-bound NPC1 [23]; then, cholesterol is moved toward ER or plasma membrane through a not fully elucidated mechanism that could involve vesicular transport [24]. This defect in cholesterol transport results in its sequestration in the LE/L compartment, which secondly decreases its content in the plasma membrane [25]. During active myelination, selected myelin proteins in OLs are transported together with cholesterol through LE/L compartment for lipid remodeling involving NPC1 and NPC2 proteins [9,26]. Thus, it is not surprising that the mutations in these proteins occurring in NPC are associated with myelin defects in patients and mouse models of disease. Pathological hallmarks of such defects will be described in the following section and the cellular mechanisms driving hypomyelination will be highlighted. 

**Table 1:** Hypomyelinating diseases connected to cholesterol metabolic processes alterations.

Main Events in Cholesterol Metabolism	Defective Protein	Impairment	Hypomyelinating Diseases	References
**Synthesis**	7-dehydrocholesterol reductase	abnormality in cholesterol production	Smith-Lemli-Opitz syndrome	[26,27]
	acyl-CoA oxidase 1 (ACOX1)	very-long-chain fatty acid (VLCFA) accumulation	Peroxisome biogenesis disorder	[28,29]
	3 b-Hydroxysteroid 8, 7-sterol isomerase	8,9-unsaturated sterols accumulation	Greenberg dysplasia, Conradi–Hunermann syndrome	[30]
**Transport**	ABCA1 transporter, HDL	No transport out cell	Tangier disease	[31]
	ABCA1, Lrp1	disrupts cholesterol homeostasis	Peroxisome biogenesis disorder	[16,29]
	NPC1, NPC2	defective cellular cholesterol transportation	Niemann–Pick disease C	[26,32]
**Accumulation**	sterol 27-hydroxylase (CYP27A1)	abnormal cholesterol accumulation	Cerebrotendinous xanthomatosis,	[33,34]
	CYP27A1, Lecithin–cholesterol acyltransferase (LCAT) enzyme, vesicle-associated membrane protein-associated protein B (VapB), and OxySterol Binding Proteins (OSBP)	higher total cholesterol and HDL and LDL levels	Sporadic amyotrophic lateral sclerosis	[35]
	beta-galactosylceramidase	galactosyl-sphingolipids accumulation	Globoid cell leukodystrophy or Krabbe disease	[36]
	arylsulfatase A (ARSA) and Prosaposin precursor (PSAP)	Sulfatides accumulation	Metachromatic leukodystrophy	[37,38]
	PLP1	co-accumulation of PLP and cholesterol	Pelizaeus–Merzbacher disease	[39,40]
	peripheral myelin protein 22 (PMP22)	co-accumulation of apoE, LRP1, and ABCA1	Charcot–Marie–Tooth, Dejerine–Sottas syndrome	[41,42,43]

## 3. Myelin Defects in NPC1

Many clinical pieces of evidence demonstrate myelin defects in patients with NPC. Analysis performed in six adult patients revealed a widespread reduction in white matter compared to age-matched controls indicative of impaired myelination [44]. In another study, diffusion tensor imaging (DTI, a magnetic resonance imaging technique used to measure the diffusion of water in white matter) revealed reduced myelin water fraction (MWF) in the white matter of two adult patients with NPC compared to 15 healthy control subjects [45], indicative of reduced myelination; in addition, the less clinically affected patient showed focal reductions in MWF in the corpus callosum in contrast to the more extensive and widespread reductions across entire fiber tracts observed in the more affected subject. Another 9-year-old patient, affected by a rapidly progressive form of NPC, showed very severe myelin reduction, the extent of which by far exceeds that of neuronal damage [32]. These pieces of evidence argue for the possibility that a correlation exists between the degree of defects in myelination and the progression of the disease, and that the therapeutic targeting of this pathological event could ameliorate the symptoms of NPC. To succeed in this aim, it is necessary to better understand the contribution of each cell type to myelin defects and the cellular mechanisms responsible for it by using animal models recapitulating the pathological hallmarks detected in patients. The NPC mouse BALB/cNctr-Npc1m1N/J, carrying a spontaneous mutation of *NPC1* causing a functional loss of NPC1 protein, is frequently used in NPC because it recapitulates many features of the early-onset human disease. Starting from the 1980s, primary dysmyelination of the cerebrum has been described in this strain [46] and further characterized by later studies. Takikita and co-workers [47] observed a markedly reduced number of mature OLs in the cerebral cortex and corpus callosum of NPC mice compared to their WT counterparts. They suggested axonal incapability in receiving myelination due to an inappropriate axon-glial interaction. A deeper insight into the molecular mechanism responsible for the reduction of mature OLs demonstrated that the absence of NPC1 protein could be responsible for the decreased expression of the myelin gene regulatory factor (MRF), a transcriptional factor critical for OLs maturation [48]. Other studies confirmed that the defects observed in the myelination process in NPC are due to an inhibition of OLs maturation rather than to a reduction in their number [49,50], and this mechanism was observed in other models of NPC; in fact, the NPC1*^nmf164/nmf164^* mouse strain (characterized by the substitution of aspartate to glycine in NPC1 protein) also shows a decrease in the expression of the myelin basic protein (MBP), a well-established marker of mature myelin, in the cerebellum (the most affected brain area in NPC) [51]. Moreover, the decrease in MBP affected either the 18.5 kDa form of the protein, which is specifically expressed by mature OLs, and the 17.5 and 21.5 kDa forms, which are specific for developing OLs. Studies using a feline model of NPC showed abnormalities also in the peripheral nervous system that was characterized by motor and sensory nerves with decreased myelin thickness and reduced axon diameter [52].

Although OLs are the main actors in the myelination process, it should be recalled that their interaction with neurons and glial cells (both astrocytes and microglia) is pivotal for myelin homeostasis [53]. To dissect the contribution of neurons and OLs to the dysmyelination process, Yu and Lieberman used a conditional mouse model in which the deletion of the NPC1 protein could be restricted to neurons or OLs [9]. Their results demonstrated that neuronal NPC1-knockout mice exhibit the dysmyelination phenotype of global null mutants and that this effect is due to a lack of maturation of OLs; in addition, the deletion of the protein in OLs (which abrogates their ability to utilize cholesterol from the endocytosis of low-density lipoprotein, LDL) causes a similar but less severe defect in myelination. The authors concluded that the entry of exogenous cholesterol inside cells and its trafficking mediated by an NPC1-dependent pathway is a key event for the formation and maintenance of CNS myelin. Moreover, their data suggest that NPC1 deficiency in neurons impairs the axonal-glial signal necessary for correct myelination. Together with neurons, microglia also play an important role in the regulation of the myelination process; among their several functions, during brain development, microglia are responsible for the recruitment of OPs and their differentiation, and the clearance of myelin debris [54,55]. Considering that an impairment of removal of myelin debris can compromise the re-myelination process after injury, it can be supposed that a defect in microglial functions can play a role in the dysmyelination occurring in NPC. Indeed, in a very elegant paper from Colombo and co-workers, the authors demonstrated that at a pre-symptomatic stage of disease BALB/cNctr-Npc1m1N/J mice exhibited hyperactive microglia characterized by enhanced phagocytic uptake and aberrant delivery of myelin into lysosomes, as demonstrated by the over-expression of the protein LGALS3, which is involved in microglial phagocytosis of myelin [56]; this caused defects in myelin turnover and the formation of intracellular lipid droplets accompanied by a strong pro-inflammatory phenotype and compromised function. Interestingly, accumulation of LGALS3 was also found in the blood serum of NPC patients, suggesting that the defects found in mouse microglia can be representative of similar impairments in patients. In addition, these results demonstrated that microglial activation is a direct consequence of the loss of NPC1 function in microglia and not an immune response secondary to degenerating neurons. In agreement with these results, Gabande-Rodriguez and co-workers analyzed microglial function in different lysosomal storage diseases [57]. Concerning NPC, in symptomatic NPC1*^nmf164/nmf164^* mice they found an increased number of microglia in the hippocampus, cortex, and cerebellum; in the latter, microglia were characterized by an amoeboid morphology and were positive to MBP staining, indicative of accumulation of myelin debris. The authors hypothesized that the protective role exerted by microglia in clearing myelin debris is corrupted by lipid overloading occurring in NPC [57].

**Figure 1:**
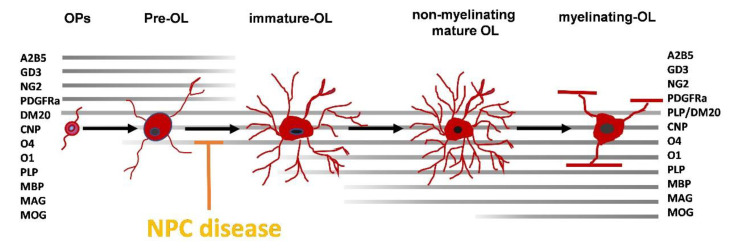
Blocking of oligodendrocyte differentiation in the NPC. Specific markers allow identifying the differentiation status of cells of the oligodendrocyte lineage from progenitors (OPs) to myelinating OLs. The stage when the maturational block due to NPC mutation occurs is indicated. The markers indicated are: A2B5 and GD3 antigens, cell surface gangliosides; NG2, cell surface chondroitin sulfate proteoglycan; PDGFRα, platelet-derived growth factor receptor alpha; DM20, a splice variant of the proteolipid protein; CNP, 2′,3′-Cyclic-nucleotide 3′-phosphodiesterase; O_4_ antigen, cell surface sulfatide; O_1_, galactocerebroside; PLP: proteolipid protein; MBP, myelin basic protein; MAG, myelin-associated glycoprotein; MOG, myelin oligodendrocyte protein.

In conclusion, these results indicate that a defective OLs maturation could trigger a myelination failure in NPC resulting in dysmyelination. In particular, this defect is caused by an impaired differentiation of pre-myelinating OLs into myelinating OLs rather than a reduced differentiation of OPs into premyelinating OLs (Figure 1).

## 4. NPC Cellular Defects Potentially Affecting OLs Differentiation

**Figure 2:**
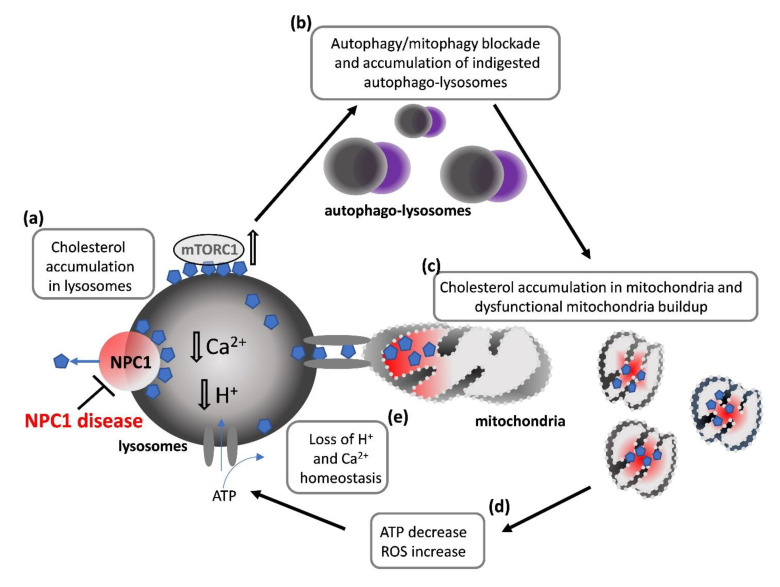
**Cellular events involved in NPC1 disease:** (**a**) cholesterol accumulation in lysosomes due to the loss of function of the cholesterol (ch) transporter NPC1; (**b**) autophagy/mitophagy blockade and accumulation of indigested autophago-lysosomes due to lysosomal accumulation of ch, ch-induced hyperactivation of the anti-autophagy complex mTORC1; (**c**) accumulation of dysfunctional mitochondria due to accumulation of ch in mitochondria and mitophagy blockade; (**d**) ATP production decrease and ROS increase by dysfunctional mitochondria; (**e**) loss of lysosomal ionic homeostasis. Blue pentagons: cholesterol.

With the intent of offering a view of the main cellular events that could also play a role in dysmyelination occurring in NPC, herein we will describe some cellular defects of NPC and discuss the potential impact on myelin-forming cells (Figure 2). Although very few data have been collected specifically in OLs, some hypotheses can be formulated about the role potentially played by such defective events in the dysmyelination observed in NPC.

### 4.1. How Lysosomal Impairment Affects Mitochondria and OLs Differentiation

Many mitochondrial functions, which are found defective in NPC, are essential for the differentiation of OPs toward mature OLs [58]. Mitochondria supply the energy required for the high metabolic rate of differentiating cells and the synthesis of large amounts of membrane components of myelin, such as cholesterol. Moreover, nuclear and mitochondrial genes coding for mitochondrial proteins are upregulated during OLs differentiation [59].

The primary trait of mitochondrial involvement in NPC is the accumulation of cholesterol in the mitochondrial membranes. Among the secondary events depicting mitochondrial functional demise observed in NPC experimental models are: a decreased mitochondrial ATP production, an increase in mitochondrial ROS and a decrease in the endogenous antioxidant glutathione (GSH), and an increase in fragmented mitochondria due to the unbalance between fission and fusion processes [60]. According to the most accepted view, cholesterol accumulation in mitochondria is due to direct contact sites between endo/lysosomal membranes and mitochondria, which in healthy conditions are involved in substrate entrance in mitochondria to fuel the Krebs cycle and in general to fulfill bio-energetic needs. The accumulation of cholesterol in mitochondrial membranes is associated with a decrease in membrane fluidity and, in turn, to the functional alteration of membrane proteins such as respiratory chain complexes and transporters [61]. Indeed, mitochondrial respiratory chain deficiency and inner membrane depolarization have been detected in mouse models of NPC [62] and fibroblasts from patients [63]; more importantly for the focus of the present review, in primary OLs modeling the maturational arrest characteristic of NPC, mitochondria showed significant morphological and functional impairments [64]. So far, a direct correlation between mitochondrial defects and dysmyelination occurring in NPC has never been investigated, but, given the pivotal role of mitochondria in regulating OLs differentiation, it would be very interesting to address this issue in future in vivo studies.

### 4.2. How Impairment in Lysosomal Functions Affects Autophagy and OLs Differentiation

Lysosomal functions are directly affected by the decrease in mitochondrial ATP production, which is crucial for the maintenance of the ionic homeostasis in lysosomes (especially of H^+^ and Ca^2+^), and the acidic pH in the lysosomal lumen [65]. In turn, acidic pH is needed for the proper activity of hydrolytic enzymes and for the control of lysosomal Ca^2+^ concentration, which is necessary for vesicle trafficking, fission, and fusion with other organelles [66]. The fusion of lysosomes with autophagosomes in autophagolysosomes is a key event to give rise to the autophagic process necessary for the clearance of unneeded materials [67]. Lysosomes are not only digestive hubs, but they also belong to a network capable to respond to the changing metabolic needs of the cell. One of the pivotal signaling mechanisms involved in the catabolic/anabolic regulation of metabolism is triggered by the nutrient-driven transfer of mammalian Target of Rapamycin complex (mTORC1) from the cytoplasm to the lysosomal membrane, where growth factors activate mTORC1 through of the PI3K-AKT pathway; once activated, mTORC1 drives metabolism toward anabolic reactions and acts as a potent inhibitor of autophagy [68]. Among nutrients capable of activating mTORC1, there are mainly amino acids, glucose, and, importantly for NPC, cholesterol [69]. 

Calcium, autophagy, and mTORC1 have been found impaired in different models of NPC, as described later on. 

A mounting body of evidence demonstrates both a decrease in lysosomal luminal Ca^2+^ concentration [5,63] and a defect of autophagy in NPC [20]; interestingly, an imbalance in the autophagic flux also in primary cultures of OLs modeling the maturational arrest occurring in NPC [64] was recently demonstrated. As concerning mTORC1, a close interaction exists with cholesterol and NPC1 protein, the first being an activator and the second a repressor of mTORC1 [68]. Mutations in NPC1 protein results in hyper-activation of mTORC1 in NPC disease and, consequently, in autophagy inhibition [68]. 

If such impairments in autophagy can play a role in the myelin defects observed in NPC has not been investigated. However, the recent observation that autophagy is essential for OLs differentiation and proper myelination [70], and that autophagy was affected in OLs modeling NPC [64], suggests a possible involvement of autophagy in NPC myelin defects. In addition, mTORC1 activity is crucial for the remyelination process, being involved both in the first phase of development of OPs from O_4_ to pre-myelinating OLs and, in later phases, characterized by increased synthesis of lipids and membrane proteins [71]; moreover, hyper-activation of mTORC1, like the one occurring in NPC, was shown to slow down remyelination [72]. Further studies investigating the role played by the autophagy defect in the maturational arrest of OLs observed in NPC are worth considering. 

## 5. Therapy Perspectives: The State of the Art

As previously shown, the current working model for the molecular mechanisms responsible for the symptomatology of NPC disease, including dysmyelination, is indisputably based on the intracellular accumulation of cholesterol. As shown in the previous section, defects in lipid transport and storage are the cause of many pathological events occurring both in neurons and in OLs. For this reason, the therapeutic interventions that have been explored so far aimed at reducing cholesterol and sphingolipids either by inhibiting their synthesis or by reducing their accumulation. Miglustat (an iminosugar drug that reversibly inhibits the first step in glycosphingolipid synthesis [73]), cyclodextrins (chelators of intracellular cholesterol), arimoclomol (an inducer of HSP70 expression that increases sphingomyelinase activity resulting in reduced accumulation of cholesterol [74]) and vorinostat (a histone deacetylase inhibitor able to increase the expression of NPC1 and reduce cholesterol accumulation [75]) are the main drugs entered in clinical trials. Very few data are present in the literature about their efficacy in reducing dysmyelination in NPC patients. The following sections will focus only on drugs that have been clinically evaluated also for their ability to ameliorate myelin defects. 

### 5.1. Miglustat 

The only drug currently approved in the EU and other countries is miglustat [22,76]. Miglustat (OGT 918, N-butyl-deoxynojirimycin) is currently marketed as Zavesca by Actelion Pharmaceuticals. Miglustat was approved in 2002 by EMA and in 2003 by FDA, as a treatment for type 1 Gaucher disease [77]. However, it can be only prescribed off-label in the US, since the FDA did not approve the drug for NPC treatment, being considered to have insufficient proof of efficacy [78], and given the adverse gastrointestinal effects [79,80]. Despite the approval, the EMA overview document of Zavesca also describes the response to the drug treatment of patients in clinical trials as limited to a slight improvement in swallowing ability and intellectual function, and to a stabilization/decrease in the rate at which symptoms worsened in about three-quarters of the treated individuals (https://www.ema.europa.eu/en/documents/overview/zavesca-epar-summary-public_en.pdf, accessed on 16 August 2021). Few data concerning the effect of miglustat on myelin defects in patients are available. It is worthy of note that one year of miglustat therapy improved fractional anisotropy (FA), a marker of axonal myelin integrity assessed by neuroimaging, in the corpus callosum of an adult NPC patient [81]. Recently, a revision of the effectiveness of miglustat was performed by assessing a range of measures and by also evaluating its effect on myelin [82]. Diffusion tensor imaging (DTI), used to measure white matter architecture and integrity, revealed that miglustat improved the FA in the corpus callosum after 1 year of therapy. Another prospective study in a cohort of 13 patients also demonstrated improvement of FA in the corpus callosum, forceps minor, and cingulate gyrus after 2 years of treatment. This study demonstrates that miglustat can improve neurological symptoms of NPC patients also by delaying the progression of the dysmyelination process [82].

### 5.2. Cyclodextrins

The reduction in the cholesterol load in cells exerted by cyclodextrins allows redistribution of the cholesterol from the late endosome/lysosome compartment to the extracellular space [83]. Even though the mechanism behind cyclodextrin functioning is not fully understood, the efficacy of the cyclic oligosaccharide 2-hydroxypropyl-β-cyclodextrin (HPβCD, a cholesterol-chelating agent) has been extensively demonstrated in NPC experimental models [83,84,85]. Preclinical studies showed promising results, such as the delayed onset of neurological symptoms and increased lifespan [22]. Consequently, many clinical trials were initiated with HPβCDs. One of these investigational products is the VTS-270 produced by Mallinckrodt Pharmaceuticals; in this phase 2/3 trial (ClinicalTrials.gov Identifier: NCT02534844), NPC patients were administered by the lumbar intrathecal route with 900–1800mg of the drug every 2 weeks. Unfortunately, in January 2021 it was discontinued from its clinical development in NPC patients because of a negative benefit/risk balance (https://www.inpda.org/wp-content/uploads/2021/01/MNK-Announcement-INPDA-Jan-2021.pdf, accessed on 16 August 2021). Another HPβCD, under the name of Trappsol^®^Cyclo™, obtained promising clinical results about its efficacy for both systemic and neurologic manifestations of NPC [86] and is going to be evaluated in a phase III study (NCT04860960), in which it is intravenously injected in patients at the dose of 2000mg/kg every 2 weeks. Since HPβCD intravenously administered has a short biological half-life [87] and does not easily pass the blood–brain barrier (BBB) [88], strategies to increase the fraction of the drug reaching the brain are currently under evaluation (PREPRINT: Carradori et al., bioRxiv 2020.07.31.230136; doi: https://doi.org/10.1101/2020.07.31.230136, accessed on 16 August 2021). At least to our knowledge, no extensive clinical evaluation of the impact of HPβCD on dysmyelination is available. However, some preclinical results demonstrated that methyl-β-cyclodextrin administration in NPC mice increased myelination [89], partially rescued lipid droplet formation, and restored the homeostasis of microglia, supporting the idea that cholesterol-lowering in NPC can be beneficial also against the perturbation of microglial functions that contribute to the dysmyelination process [56].

## 6. Pharmacological Advance in the Development of New Targets: Preclinical Studies

Despite the many potential molecular targets for the improvement of myelin defects in NPC, very few drug candidates have entered in clinical trials. Indeed, finding an effective cure for NPC is extremely challenging for many reasons: (i) treatments should be efficacious in both the brain and peripheral organs (i.e., liver and spleen); (ii) early diagnosis is unusual and thus, the therapy initiates after neuronal death and hypomyelination have (sometimes irreversibly) progressed; (iii) being an ultra-rare disease, finding a sufficient number of patients with similar disease severity is very difficult.

Nevertheless, many efforts in the field are in progress to find new “druggable” targets, such as replacement therapies aimed at transferring the *NPC1* gene in mice, which indeed improved behavioral abnormalities and increased their lifespan [90,91,92]. Neither these studies nor others evaluated the impact of the treatment on myelin defects. Nonetheless, N-Acetyl-L-Leucine, a derivative of the branched-chain amino acid leucine, significantly reduced neuroinflammation and lipid storage in NPC1-/- mice [93]. More interestingly for the focus of this review, a protective effect of N-Acetyl Cysteine was observed in OPs both in vitro, in terms of suppression of apoptosis and in vivo, as attenuation of motor impairment and white matter dysmyelination in the corpus callosum [94]. A Phase II study (NCT03759639) is currently assessing the safety and efficacy of N-Acetyl-L-Leucine (IB1001) for the treatment of NPC. 

Another potential drug tested in preclinical models of NPC is lovastatin, an inhibitor of 3-hydroxy-3-methylglutaryl-coenzime A (HMG-CoA) reductase, which was evaluated for its ability to induce differentiation of OLs derived from a mouse model of NPC; the results showed that it inhibited cholesterol synthesis, reduced its accumulation in endo-lysosomes, and increased the maturation of NPC OLs [49]. 

The neurosteroid allopregnanolone was tested as well in mouse models of NPC [95]. The rationale for testing allopregnanolone resides in the observation that sequestration of cholesterol inside cells would alter neurosteroidogenesis and contribute to the neuropathology of NPC [96]. Indeed, allopregnanolone levels are found decreased in NPC [95]. The beneficial effects of allopregnanolone on dysmyelination, alone or in combination with HPβCD and miglustat, have been extensively investigated in NPC mice [95,97,98]. Specifically, the administration of allopregnanolone solubilized in HPβCD to NPC mice was effective in delaying clinical onset, extending lifespan, and reducing ganglioside accumulation [95]; moreover, this treatment also normalized myelin content in the corpus callosum and hippocampus measured by DTI [89]. However, considering that in the above-mentioned studies allopregnanolone was dissolved in HPβCD that as such is beneficial in NPC, it is possible that the myelination process promoted by the treatment is not due to allopregnanolone but rather to its vehicle HPβCD. Indeed, Davidson found that the combination therapy of miglustat with allopregnanolone dissolved in HPβCD ameliorated the NPC disease, but also the vehicle (HPβCD) provided significant benefits; on the contrary, allopregnanolone without HPβCD did not appear beneficial [99]. Similar results were obtained also by Liu and co-workers who found that HPβCD prolonged the average life of NPC mice (>108 days), but the addition of allopregnanolone had no additive effects [84]. 

Among new therapeutic targets under preclinical evaluation in NPC models, our group focused on the neuromodulator adenosine, which will be described in detail in the following section.

## 7. Role of Adenosine and Adenosine A_2A_ Receptors in NPC

Adenosine is a nucleoside ubiquitously distributed throughout the body and a paracrine homeostatic modulator of different cellular functions. In the CNS, adenosine plays an important role in controlling synaptic plasticity, cognition, sleep, motor function, and neuronal survival [100]. Its levels are finely tuned by the orchestrated action of enzymes and transmembrane transporters (ENTs) that ensure the physiological level of extracellular adenosine necessary to exert its proper receptor-dependent and -independent pathways [101]. In particular, four G-protein-coupled receptors are mainly involved in its signaling: A_1_, A_2A_, A_2B_, and A_3_ [102,103]. Among them, the A_2A_ receptors (A_2A_Rs) seem to have particular relevance in NPC. These receptors, which are mainly coupled to Gs resulting in AC-cAMP-PKA pathway activation, play a major role in the brain, being effective modulators of neuronal damage in various pathological situations, and both their activation and blockade are known to result in being neuroprotective in different experimental conditions, probably involving multiple concerted actions [104,105,106,107]. Although A_2A_Rs are most abundant in the striatum, they are also present in the hippocampus, where they finely modulate synaptic transmission and excitotoxicity [108], and in the cerebellum, where their function is still poorly characterized, being these brain areas, both affected in NPC.

Furthermore, the importance of keeping the levels of brain adenosine to an appropriate physiological level is demonstrated by the observation that a deregulated signaling was found in many neurodegenerative diseases such as Alzheimer’s disease (AD), epilepsy, and Huntington’s disease (HD), and the enhancement of adenosine levels in these pathological conditions was found beneficial [109,110]. 

Adenosine plays a pivotal role also in modulating the myelination process as demonstrated by its ability to affect migration, proliferation, and maturation of oligodendroglial cells. As comprehensively described in previous reviews, its action on OLs depends on the receptor subtype that is stimulated [111]. Of particular interest for the present review is the role played by the adenosine receptor A_2A_ both in the myelination process and in the NPC pathology. The group of Coppi and co-workers characterized the function of A_2A_R in OPs by demonstrating that in vitro stimulation of the receptors with the selective agonist CGS21680 delayed their differentiation into OLs without affecting cell viability [112]; in addition, the A_2A_R antagonist SCH58261 in zebrafish larvae induced OPs migration from motor exit point in the transition zone [113]. These results demonstrate that A_2A_R can modulate both the differentiation and the migration of OPs.

Concerning NPC, different evidence indicates an imbalance of the adenosine signaling: first, a reduced level of adenosine has been consistently shown in the brain of NPC1-/-mice [114] that could be responsible for the impaired synaptic plasticity observed in this model and, as a consequence, for their cognitive deficits. As a consequence of adenosine reduction, the signaling mediated by its receptors could be impaired; in fact, different in vitro and in vivo studies in our lab demonstrated that the stimulation of the A_2A_ receptors was able to restore a normal phenotype in NPC cellular models. We first demonstrated that in fibroblasts from NPC1 patients the A_2A_R stimulation by the agonist CGS21680 restored lysosomal calcium content, mitochondrial membrane potential (mMP), and cholesterol distribution [63]. The use of fibroblasts from NPC patients is relevant but, considering the major involvement of the CNS in NPC pathology, it is important to test drugs also in cellular models representative of the cellular abnormalities affecting neuronal and glial cells. For this reason, we performed experiments in neuronal and oligodendroglial cell lines of human origin and we induced the NPC1 phenotype by small-interference RNA. As we had already observed for fibroblasts, also in “CNS cell lines” the A_2A_R stimulation by CGS21680 was effective in reducing cholesterol accumulation and in normalizing mitochondrial membrane potential [115]. These results paved the way for the next in vivo studies, in which NPC1-/-mice were treated with the compound T1-11 that can weakly stimulate A_2A_R and increase the level of adenosine in the brain by inhibiting its transporter ENT1. The drug significantly ameliorated the cognitive deficits of mice, reduced Purkinje neuron loss and sphingomyelin accumulation in the liver, and extended their survival [116]. 

**Figure 3:**
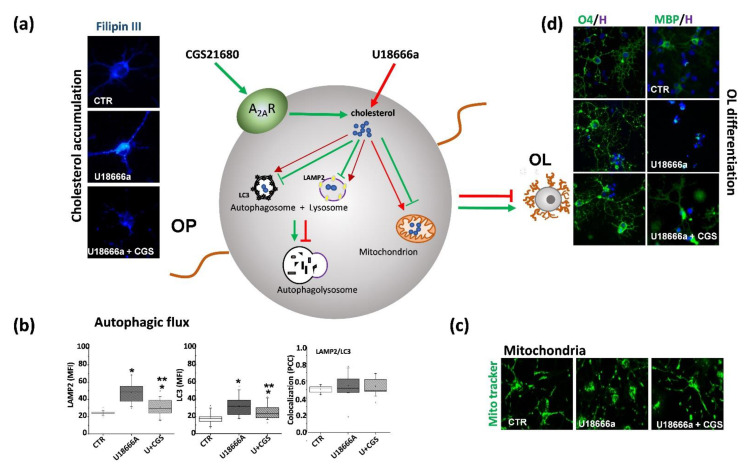
**CGS21680 restores oligodendrocyte functions and differentiation in the NPC-like pharmacological culture model.** The A_2A_R agonist CGS21680 counteracts the effects induced by the cholesterol transport inhibitor U18666A, known to induce an NPC-like phenotype: (**a**) by reducing the accumulation of cholesterol, as shown by the cholesterol probe Filipin III; (**b**) by restoring the autophagic flux, as evidenced by normalization of LC3 and LAMP2 markers (* *p* < 0.05 vs CTR; *** *p* < 0.05 vs U18666A); (**c**) by normalizing mitochondrial morphology and membrane potential; (**d**) by counteracting the maturation arrest and favoring OLs differentiation toward cells responsible for myelin formation. Green and red lines illustrate CGS21680 and U18666A effects, respectively; arrows and truncated lines indicate positive and negative effects, respectively.

Given the modulatory role of A_2A_R in the differentiation of OLs and considering that a delay in oligodendroglial maturation seems to be the cause of the dysmyelination pathology in NPC disease, we decided to analyze the impact of its stimulation on OPs maturation [64]. To this aim, we exposed primary cultures of OPs to U18666a, an inhibitor of cholesterol transport usually used to induce an NPC-like phenotypes in vitro [80]. As expected, U18666a induced typical features of NPC1 phenotype such as intracellular accumulation of cholesterol, abnormal mitochondrial depolarization, and impaired autophagy. Moreover, it caused a maturational arrest of OPs as demonstrated by the decrease in the percentage of O_1_ (immature OLs) or MBP (non-myelinating mature OLs)-positive cells. The treatment with CGS21680 overcame the maturation arrest (demonstrated by the increase in the percentage of O_4_, O_1_ and MBP expressing cells) induced by U18666a and restored the complex, arborized morphology of cells [64]. Interestingly and in agreement with Coppi and co-workers, in control cultures (i.e., in absence of U18666a) CGS21680 induced an arrest in OLs differentiation as indicated by the decrease in the percentage of O_1_ and MBP expressing cells [112]. The opposite effect of A_2A_R stimulation in healthy and in NPC1 cells could indicate a profound functional change of this receptor induced by intracellular cholesterol accumulation and by its consequent depletion in the membrane. Indeed, a reduction in membrane cholesterol concentration such that occurring in NPC [117] can inhibit the activity of A_2A_R as indicated by the reduction in cyclic adenosine monophosphate (cAMP) production [118]. Such a “double-faced effect” of A_2A_R stimulation has been already observed in other pathological conditions such as in HD; in fact, in HD mice (the R6/2 model) CGS21680 potentiated the toxicity induced by NMDA receptor stimulation in WT mice but attenuated it in HD littermates [119]. Thus, we can hypothesize that in physiological conditions, an overactivation of the receptor can result in a maturation arrest of OLs, but the lysosomal entrapment of cholesterol caused by U18666a, can reduce its tonic activation; consequently, receptor stimulation exerted by CGS21680 can restore the basal signaling necessary for the proper maturation of OLs. Together with the beneficial effect observed on differentiation of OLs, our data also demonstrated that the stimulation of A_2A_Rs reduced intracellular cholesterol accumulation, mitochondria abnormalities and rescued from the unbalanced autophagic flux induced by U18666a in OPs (Figure 3) [64].

In conclusion, these data suggest that the stimulation of A_2A_R could represent a promising therapy for NPC disease because it can simultaneously impinge on many pathological events such as cholesterol accumulation, mitochondrial dysfunction, autophagy, and OLs differentiation.

## 8. Conclusions

Preclinical research in NPC is at an earlier stage relative to other neurodegenerative disorders, struggling with the complexity of the molecular abnormalities observed in NPC experimental models and the consequent poor translation to the clinical setting [120]. Looking on the bright side, the knowledge of the molecular pathways contributing to the NPC pathophysiology is rapidly increasing, and considerable knowledge has emerged from the preclinical studies conducted so far, in vitro and in vivo, in NPC models. Like for all the orphan disorders, clinical research in NPC is a challenging task, since its rarity does not allow to conduct large clinical trials. The difficulties are also related to the lack of early diagnosis of NPC in many young patients, and to the fact that the treatment should be effective in both the brain and visceral organs. For these and other reasons, no effective treatment, except for miglustat, is currently available for the NPC disease. Among the different outcome measures used in NPC preclinical and clinical studies, dysmyelination is one of the newest, and more interesting, phenotypes considered (see Table 2 for a summary), since it is a feature of NPC in both patients and animal models [44]. The increase in adenosine levels and A_2A_R stimulation could represent therapy perspectives in NPC, considering their beneficial effects on dysmyelination.

## Figures and Tables

**Table 2:** Drugs evaluated in NPC and their effects on dysmyelination.

Drug	Main Mechanism of Action	Phase (or Clinical Use if Applicable)	Effect on Dysmyelination (Preclinical)	Effect on Dysmyelination (Clinical)	References
Miglustat	Glycosphingolipid synthesis inhibition	Approved by the EMA for clinical use in NPC Prescribed off-label in the US	no data available	Improvement of fractional anisotropy (FA)	[81,82]
Hydroxypropyl-β-cyclodextrin (HPβCD)	Cholesterol chelation and redistribution	phase III, ongoing	Increased myelination, rescued lipid droplet formation, restored homeostasis of microglia	Improvement in fine and gross motor functions, and swallowing	[56,85,88]
N-Acetyl-L-Leucine	Neuroinflammation Reduction	phase II, ongoing	Protective towards oligodendrocyte progenitor cells in models of neonatal hypoxic-ischemic encephalopathy (HIE)	no data available	[93]
Lovastatin	Inhibitor of 3-hydroxy-3-methylglutaryl-coenzime A (HMG-CoA) reductase	none	Reduced cholesterol accumulation and increased the maturation of NPC OLs	no data available	[49]
Allopregnanolone	Neurosteroid deficient in NPC mice	none	In NPC mice, allopregnanolone solubilized in HPβCD delayed clinical onset, extended lifespan, reduced ganglioside accumulation, normalized myelin content	no data available	[88,94,95,96,97]
CGS21680	Adenosine A_2A_ receptor agonist	none	Overcame the OP maturation arrest, restored the morphology of cells, reduced cholesterol accumulation, mitochondria abnormalities, and protected OP from the unbalanced autophagic flux	no data available	[64]

## Data Availability

Not applicable.

## Abbreviations

ABC	ATP-binding cassette
ACOX1	acyl-CoA oxidase 1
ApoE	Apolipoprotein E
ARSA	Arylsulfatase A
BBB	Blood–brain barrier
CNS	Central nervous system
CY27A1	Sterol 27-hydroxylase
DHC	Dehydrocholesterol
DHCR	Dehydrocholesterol reductase
HDL	High-density lipoprotein
HMG CoA	Hydroxymethyl-glutaryl-CoA reductase
HSP70	Heat Shock Protein 70
LCAT	Lecithin–cholesterol acyltransferase
LDL	Low-density lipoprotein
LRP1	Low-density lipoprotein receptor-related protein-1
MAG	Myelin-associated glycoprotein
MBP	Myelin basic protein
mTORC1	Mammalian Target of Rapamycin complex 1
NPC1	Niemann-Pick C1 Protein
NPC2	Niemann-Pick C2 Protein
OL	Oligodendrocyte
OP	Oligodendrocyte precursor cell
OSBP	OxySterol Binding Proteins
PLP	Proteolipid protein
PM	Plasma membrane
PMP22	Peripheral myelin protein 22
PNS	Peripheral nervous system
PSAP	Prosaposin precursor
RE	Endoplasmic reticulum
VapB	Vesicle-associated membrane protein-associated protein B
VLCFA	Very-long-chain fatty acid
